# Assessing Mechanisms Underlying the Sharing of Official and Unofficial Information during a Pandemic

**DOI:** 10.3390/ijerph182413298

**Published:** 2021-12-17

**Authors:** Stephanie Jean Tsang, Xinyan Zhao, Yi-Ru Regina Chen

**Affiliations:** 1 Department of Communication Studies, Hong Kong Baptist University, Kowloon Tong, Hong Kong, China; yrchen@hkbu.edu.hk; 2 Hussman School of Journalism and Media, University of North Carolina, Chapel Hill, NC 27514, USA; ezhao@unc.edu

**Keywords:** anger, anxiety, information sharing, political orientation, unofficial information

## Abstract

The COVID-19 disease outbreak has seen mixed information flows comprising top-down communication from health authorities to the public and citizen-to-citizen communication. This study aimed to identify mechanisms underlying the sharing of official versus unofficial information during the outbreak. Survey findings based on a nationally representative U.S. sample (N = 856) showed that individuals’ predispositions affected their information consumption and affective experiences, leading to distinct types of information-sharing behaviors. While anger toward the U.S. government’s outbreak response was directly associated with unofficial information sharing, anxiety was directly associated with official information sharing. These findings enhance our understanding of the propagation of different kinds of pandemic information and provide implications for public education on information verification based on source authoritativeness.

## 1. Introduction

Research on the role of Information and Communication Technologies (ICTs) in significant events is gaining traction [1]. Specifically, during a pandemic, ineffective communication between authorities and citizens can hinder the prompt adoption of preventive measures, impairing community recovery. How information is disseminated both between officials and citizens and among citizens requires further research [1], especially given the explosion of risk campaign messages, user-generated content, and complex dynamics of (mis)information dissemination are enabled by ICTs and social media [2,3]. Therefore, it is vital to understand how to encourage the sharing of official information among citizens, especially information generated by agencies that are staffed with science personnel to review health-related data in a scientific manner.

ICTs have afforded convenient access to and dissemination of both official and unofficial information during the pandemic [4]. Information from an authoritative source (i.e., the World Health Organization (WHO), Centers for Disease Control and Prevention (CDC), National Institute of Health (NIH), and Food and Drug Administration (FDA)) is defined as official information in this study. A better understanding of official information sharing allows more timely and proactive responses for enhancing the efficiency of crisis management. On the other hand, unofficial information includes pandemic information from unofficial sources such as online forums, ordinary citizens, or alternative news. Although unofficial information is less likely to be verified than official information, these sources do not necessarily contain false information, and similarly, an official source does not necessarily mean that the information is accurate. Since only approximately 20% of U.S. news consumers seek the latest information about COVID-19 from CDC, NIH, and FDA [5], this study aimed to uncover the mechanisms behind the sharing of official versus unofficial information. Such an understanding would allow health authorities to facilitate the dissemination of authoritative information and enhance the effectiveness of their crisis communication.

Given that health crisis communication is critical in times of a pandemic, this study explored the dynamics of audiences’ predispositions, affective experiences, information seeking, and sharing of communication sources (e.g., public officials, family, friends, news organizations, etc.) by adopting the O-S-O-R model [6]. The findings can shed light on how to improve communication practices, taking both top-down practices and peer communications into account simultaneously. While models predicting sharing of information do exist, this study considered the mechanisms underlying the sharing of unofficial information alongside the sharing of official information. More importantly, there has never been a time when health communication has been so politicized, such that Pew data have shown that Americans form divergent attitudes towards organizations such as the WHO and CDC, with Democrats trusting CDC and WHO and Republicans distrusting both organizations [7,8]. Our study examined how individuals’ predispositions, types of COVID-19 information-seeking behaviors, and emotions toward the pandemic, taken together, affect their sharing behaviors of official and unofficial information about the pandemic, adopting the O-S-O-R model. The findings can offer insights that will help health organizations to meet the information needs of users by promoting official content on the risks of the pandemic.

### 1.1. Crisis Information Sharing: Official vs. Unofficial Information

Information sharing refers to the “practice of giving a defined set of people access to news content via social media platforms, as by posting or recommending it” [9] (p. 2) During the COVID-19 pandemic, information exchanges and expressions of opinion online have made social media important channels for information sharing. Often, governmental agencies provide important guidance for the public and news media regarding appropriate responses [10], and citizens typically rely on government instructions to decide on preventive behaviors [11]. In this pandemic, the global community has depended on the WHO to coordinate the response. In the United States, several federal health agencies representing the national government’s official voice have been responsible for containing the pandemic, including the CDC, NIH, and FDA.

Although Vraga and Bode [12] stated, “expert consensus can sometimes provide clearer boundaries between what is accurate and inaccurate” (p. 137), “experts” and “evidence” can be subjective and difficult to define. Expert consensus is not always available, even among health authorities. During the initial stage of the COVID-19 pandemic, for example, health and science experts held different opinions about whether and when one should wear a facemask. Nevertheless, we can expect a wide consensus in the scientific community to underpin solutions provided by major health authorities (CDC, NIH, and FDA) in comparison to information not released by these identified, official sources.

In fact, Helsloot and Grosnendaal [13] showed that information published by governments becomes buried under citizens’ tweets during impactful emergencies. To enhance the effectiveness of authorities’ communication with citizens and facilitate the spread of official, authoritative information, this study examined the antecedents and mechanisms accounting for citizens’ engagement with official information sources. Such an understanding could even provide important implications for media literacy programs by providing media users with knowledge and efficacy to verify information based on source authoritativeness [14]. In fact, while substantial scholarship has been devoted to studying rumor transmission during disasters [15], less is known about the transmission of unofficial information and the withholding of official information in a health-related crisis context.

### 1.2. The O-S-O-R Model

**Figure 1:**
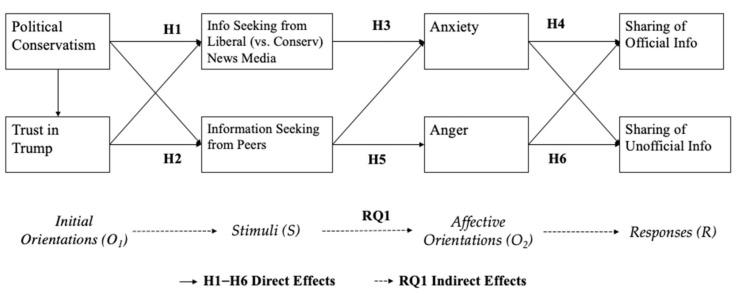
Theoretical model (O_1_-S-O_2_-R).

The O-S-O-R model is an effective framework for explicating complex social cognition processes as it recognizes an individual’s preexisting orientation (O_1_) and the mediating effects of media content exposure (S) on personal–psychological factors (O_2_) [16]. The model has been widely employed in political communication contexts and recently applied to the health communication context [17,18]. Political communication literature guided by the model often theorizes the individual’s political orientation as the O_1_ variable and political participation or efficacy as the response (R) variable. Previous health communication studies using the O-S-O-R model have examined audience perceptions (e.g., concerns, perceived severity, and perceived susceptibility) and knowledge of a health issue as the preorientation variable, attitudes toward the issue or the health practitioners and agencies involved as the O_2_ variable, and the desired behavioral change with regard to the issue as the response variable. Adopting the O-S-O-R model, this study examined how audiences’ political orientation and trust in Trump (O_1_) affected their COVID-19 information seeking from news media or peers (S) and their emotions toward the pandemic (O_2_), which further led to their information-sharing behaviors (R). In other words, multiple predictors are expected to act on information-sharing behaviors simultaneously (see Figure 1).

### 1.3. Initial Orientations (O_1_)

The first orientation, O (O_1_), is often conceptualized as the preexisting structural and motivational characteristics that media users possess [19,20]. This study included two situational characteristics of Americans related to the COVID-19 pandemic: political orientation and trust in Donald Trump, the president of the United States from 2017 to 2021. Because demographics are likely to influence media behaviors [21], other orientation variables such as age, gender, education level, and income were included as control variables in our model.

### 1.4. Political Orientation

Political orientation is often treated as an antecedent in O-S-O-R models. Given the politicized nature of the COVID-19 pandemic [22], political orientation is likely to influence individuals’ evaluations of the health crisis and, in turn, their information-seeking and -sharing behaviors. During the crisis, Republicans have been found to believe that the death toll was lower and social distancing was less necessary than Democrats [22]. Although most official agencies called for social distancing measures, such as cancelling public events and working from home [23], Republicans were found to believe less in these measures than Democrats [22]. Overall, Republicans were much more likely than Democrats to believe that the media were exaggerating the risks of COVID-19 [24]. Research has consistently confirmed that politically conservative Americans tend not to perceive the COVID-19 pandemic as a health priority, and thus are less motivated to seek information from liberal news media than conservative news media. This is because liberal news media typically portray the pandemic as a critical public health threat and suggest the importance of preventive actions. Since conservatives tend to believe that the media has exaggerated the risks of COVID-19 [25], they were anticipated to seek less information from attitude-conflicting sources (i.e., liberal news media) and more information from alternative information sources, such as people from their surroundings (i.e., family and friends). Hence, we hypothesized the following:

**H1:**  *Political conservatism relates (a) negatively to seeking information from liberal relative to conservative news media (i.e., liberal (vs. conservative) news media) and (b) positively to seeking information from peers.*

### 1.5. Trust toward Donald Trump

Trust in the sitting U.S. president, which is closely related to political orientation, can also play a role in information-seeking behaviors. Given that some conservative politicians downplayed the threat of COVID-19 [26], Americans who trusted Trump might not consider the disease a health priority and thus might turn to like-minded information to avoid exposure to information that does not align with their beliefs. In fact, the observed partisan differences in distancing were associated with subsequently higher COVID-19 infection and fatality growth rates in pro-Trump counties. Further, support for Trump was found to relate positively to individuals’ beliefs on whether the COVID-19 threat was exaggerated [27]. Together with the fact that liberal news media are likely to disapprove of the Trump administration’s handling of the COVID-19 outbreak, as well as the tendency for individuals to seek information consistent with their personal beliefs [28], those who trust Trump might turn from liberal news sources to alternative ones and believe in information that supports their personal political attitudes [29], and vice versa among those who distrust Trump.

**H2:**  *Trust toward Donald Trump relates (a) negatively to seeking information from liberal relative to conservative news media and (b) positively to seeking information from peers.*

### 1.6. Stimuli (S)

Information seeking is a key correlator of information sharing [30]; information sources have been found to generate different levels of intention to seek and share information during disasters [31,32], and our research treated information seeking both from liberal (vs. conservative) news media and from peers as stimuli (S). According to Glenski et al. [33], an article headline alone may prompt an individual to share and endorse a news article. Just as news consumption can foster online discussions and encourage civic engagement [34], we expected liberal (vs. conservative) media consumption to enhance people’s knowledge on COVID-19 risks as well as preventive measures [23], encouraging them to engage further in the issue, including sharing information. As shown in a Lee et al. [35] study, exposure to stomach cancer information from the media was found to impact the processing of that information. Hence, we expected issue-specific media use to play a role in the model predicting information-sharing behaviors.

Given that liberal news media are likely to criticize the work done by Donald Trump, liberal (vs. conservative) media consumers were expected to be exposed to more official information published by CDC, NIH, and FDA [36]. Being exposed to more information on COVID-19, liberal (vs. conservative) media consumers might be more aware of the potential risks of COVID-19. Similarly, the more they talked to people around them about the pandemic, the more they would be informed about COVID-19 risks and influences. Hence, information seeking, in general, is likely to expose readers to more information, official or not, and such information-seeking behavior was expected to nurture the circulation of both official and unofficial information. This expectation resembles findings on the positive effect of individuals’ search for information and their participatory behaviors [37], such as sharing information on their social networks [38]. In the current study, we specifically expected media use to impact information sharing via two affective routes, anxiety and anger.

### 1.7. Affective Orientations (O_2_) and Responses (R)

The second orientation, O (O_2_), refers to the cognitive or affective state of the audience which leads to the audience’s responses (R) to the media message, such as sharing the message [6]. The risk and crisis communication literature has identified two affective factors in the health-related crises that were examined in this study as the O_2_ variables: anxiety and anger. They can determine the type of information shared. Even though anxiety and anger are both regarded as negative emotions, they have been found to drive different attitudes and behaviors [39]. Research has shown anger to be a form of aversion, closely related to emotions such as disgust and contempt [40]. Anxiety, on the other hand, has been shown to be more closely related to emotions such as depression [40]. According to Marcus et al. [39], anxiety is likely to be driven by one’s surveillance system when threatening circumstances are identified, while anger “arises when familiar threats impede our way” (p. 203). The two are therefore distinct emotions and were anticipated to invoke different information-sharing behaviors.

The rationale for including these two specific emotions in our model is that anxiety and anger are commonly found to associate with information-seeking intentions and behaviors [41,42]. Moreover, since the two emotions have been shown to invoke divergent outcomes, with anxiety motivating avoidance and anger motivating aggression [43], it was likely that people would develop diverse information sharing behaviors during COVID-19. The majority of citizens did not have first-hand experiences with COVID-19 but did consume pandemic information through the consumption of information from news media and peers. While liberal news media outlets tend to cover COVID-19 as a serious public health threat, seeking information from peers is likely to result in similar affective reactions, as learning more about a pandemic at face value is expected to increase related risk perceptions.

#### 1.7.1. Anxiety

Anxiety is often associated with uncertainty and a lack of personal control [44], which is to be expected during a pandemic. According to Valentino et al. [45], findings on the motivational effects of anxiety are mixed. In political communication literature, Berger [46] found a positive relationship between anxiety and information sharing, but Lerner and Keltner [47] found anxiety to be associated with withdrawal, which discouraged engagement. In risk-communication literature, Jin et al. [48] found anxiety to be associated positively with disaster information-sharing behaviors, with individuals seeking not only to express their emotions but also to cope with their stress by communicating with people they know intimately. Indeed, people often experience anxiety when they face an imminent threat [49], leading them to adopt protective behaviors [50], and scholars have generally found a close relationship between risk perception and anxiety [51]. In the pandemic context, anxiety should drive sharing of both official and unofficial information, as both allow people to cope with their stress, connect with those who might provide beneficial information and social support, and determine useful protective behaviors for the potentially threatening circumstances. As anxiety should not prompt differences in information sharing, we assumed that anxiety is positively related to both official and unofficial information sharing. Further, people with more exposure to information tend to have more opportunities to share; hence, we hypothesized:

**H3:**  *Information seeking through (a) liberal relative to conservative news media and (b) peers relates positively to anxiety.*

**H4:**  *Anxiety relates positively to sharing both (a) official and (b) unofficial information.*

#### 1.7.2. Anger

Anger is often experienced when there is a target [50] or an individual or organization to blame [49]. In the COVID-19 pandemic, for example, the public has blamed the government for doing too little to prevent the crisis and/or to mitigate its negative impact. Scholars have also found anger to be an approach emotion associated with mobilization and behavioral actions [50]. According to Hasell and Weeks [52], an easy way to channel anger is to share information; if people are angry with a target, they are likely to share negative information to discredit or punish that target. Health crisis literature shows that an individual’s anger at a health provider can drive his or her acceptance and dissemination of unofficial information [53]. For example, Han et al. [54] found that in South Korea, the public’s anger was positively associated with their acceptance of COVID-19 rumors, while in China, anger-inducing rumors about the pandemic (e.g., how it was managed) triggered people’s dissemination of the rumors on social media [55]. In this study, we expected that if people were angry with the government’s response to the pandemic, they would share unofficial but not official information about the pandemic with others to challenge the government’s action.

**H5:**  *Information seeking through (a) liberal relative to conservative news media and (b) peers relates positively to anger.*

**H6:**  *Anger relates (a) negatively to sharing official information and (b) positively to sharing unofficial information.*

### 1.8. Mediating Mechanisms

The study of mediation is important to the assessment of direct effects with considerations of contextual and situational factors, as the mere inclusion of direct effects is often insufficient to fully explain human behaviors [16,56]. Hence, the proposed model recognizes the mediating effects of an individual’s pre-existing orientation on the consequences of media content exposure [16]. In particular, anxiety and anger can differentially mediate the effects of people’s predispositions on their distinct information-sharing behaviors. For example, due to the attributional nature of anger [57], those of certain political orientations can share unofficial information out of anger (but not anxiety). Moreover, those of certain political orientations may be driven by anxiety to share official information [58]. Indeed, liberal media users were found to relate positively to preventive behavior engagement [59]. Given that no prior studies have used the O-S-O-R model to distinguish between official and unofficial information sharing, a research question was posed to address the mediations among the different components for official versus unofficial information sharing.

**RQ1:**  *To what extent do affective orientations (O_2_) and stimuli (S) mediate the relationship between audiences’ predispositions (O_1_) and responses (R)?*

## 2. Materials and Methods

This research utilized survey data collected from 21 to 26 April 2020, within the three months after the WHO and the Trump administration declared COVID-19 a public health emergency [60]. During this time, people were recommended to wear face masks and perform social distancing, and states and territories were starting to issue mandatory stay-at-home orders [60]. An online panel of respondents were recruited through Qualtrics (*N* = 856). To ensure a sample that closely resembled the demographic distribution reported by the U.S. Census Bureau, quota sampling in terms of gender, age, and education was performed. Given that the recruitment of female participants aged 65 or above and holding less than a high school degree was difficult, the quotas for participants holding less than a high school degree were reduced to 6% from 13% and distributed equally across the other education groups. A sample of 856 was recruited. The sample was comparable with the U.S. national population in terms of gender (51.5% female), age (range: 18–86, *M* = 46.42, *SD* = 17.29), and education (range of scale: 1–8, *M* = 3.44, *SD* = 1.62, *Mdn* = some college). The average household income reported by the sample was between $50,001 and $60,000.

### 2.1. Measurement

Most items used in this research were adopted from previous studies on crisis and risk communication [48,61,62]. Items for sharing of official and unofficial information were constructed based on the presence of various sources in the COVID-19 pandemic.

#### 2.1.1. Political Conservatism

On a seven-point scale, respondents were asked whether they considered themselves to be liberal (1), conservative (7) or somewhere in between (*M* = 4.02, *SD* = 1.73).

#### 2.1.2. Trust toward Donald Trump

This was measured by the question, “How much do you trust President Donald Trump?” on a seven-point scale from 1 (not at all) to 7 (extremely) (*M* = 3.72, *SD* = 2.34).

#### 2.1.3. Information Seeking from Liberal Relative to Conservative News Media

Information seeking from liberal news media was measured by asking respondents, “How often have you looked for information regarding COVID-19 from the following sources? (a) New York Times, (b) ABC News, (c) CBS news, and (d) NBC news.” The scores of the four items were averaged to measure liberal news media information seeking (*M* = 3.49, *SD* = 1.62, Cronbach’s alpha = 0.82). Information seeking from conservative news media was measured by asking respondents, “How often have you looked for information regarding COVID-19 from Fox News?” (*M* = 3.65, *SD* = 2.32). Dividing the liberal media consumption score by the conservative media consumption score, we derived the relative score (*M* = 1.55, *SD* = 1.40).

#### 2.1.4. Peers’ Information Seeking

Information seeking from peers was measured by asking respondents, “How often do you seek information regarding COVID-19 from (a) family, (b) friends, (c) coworkers?” The mean of these items was calculated to represent peers’ information seeking (*M* = 4.19, *SD* = 1.80, Cronbach’s alpha = 0.81).

#### 2.1.5. Anxiety

To measure anxiety, respondents were asked to what extent COVID-19 made them feel the following emotions: (a) anxious, (b) worried, and (c) concerned from 1 (not at all) to 7 (extremely). The average score of the three items was calculated (*M* = 5.09, *SD* = 1.63, alpha = 0.90).

#### 2.1.6. Anger

To measure anger, respondents were asked to indicate the extent to which they disagreed (1) or agreed (7) with the following statements with respect to their emotions toward the United States governmental emergency responses in the COVID-19 pandemic: (a) “I feel angry”, (b) “I feel irritated”, and (c) “I feel annoyed”. The average of the three items was obtained to form the anger measure (M = 4.38, SD = 1.84, alpha = 0.97).

#### 2.1.7. Sharing of Official Information

Sharing of official sources was measured on a seven-point scale ranging from 1 “never” to 7 “frequently” by asking respondents how often they had shared information regarding COVID-19 from the following sources: (a) the Centers for Disease Control and Prevention (CDC), (b) the National Institutes of Health (NIH), and (c) the Food and Drug Administration (FDA). The average of all four items was calculated (*M* = 3.24, *SD* = 1.92, alpha = 0.93).

#### 2.1.8. Sharing of Unofficial Information

Sharing of unofficial sources was measured on a seven-point scale ranging from 1 “never” to 7 “frequently”. Respondents indicated how often they had shared COVID-19 information to other people that was (a) attributed to an unfamiliar source, (b) not attributed to a certain source, (c) not attributed to a credible source, (d) not issued by an official source, and (e) unofficial, from someone on the Internet. The average of all five items was calculated (*M* = 2.32, *SD* = 1.52, alpha = 0.93).

#### 2.1.9. Covariates

Demographic covariates included age, gender, education level, race/ethnicity (white vs. nonwhite), and income level. Additional covariates included (1) issue involvement, i.e., the extent to which respondents perceived the pandemic as important and relevant (*M* = 6.02, *SD* = 1.60, Cronbach’s alpha = 0.87); and (2) social media information seeking, i.e., the tendency that respondents sought COVID-19 information from social media platforms including Facebook, Instagram, Twitter, TikTok, and Snapchat (*M* = 2.44, *SD* = 1.62, Cronbach’s alpha = 0.87).

### 2.2. Analytical Schemes

We tested our path model using the R Lavaan Package [63]. A construct with multiple indicators was identified by a composite based on the average scores of all items. To adjust for the measurement error, the error variance of the multi-item construct was fixed at (1-Cronbach’s α) times the indicator’s variance [64]. Note that the covariates were regressed on all endogenous variables. In the path model (Figure 1), the exogenous variables were political conservatism and trust toward Donald Trump. The first layer of endogenous variables included information seeking from liberal relative to conservative news media and peers’ information seeking. The second layer of endogenous variables included perceived anxiety and anger. The last layer of endogenous variables included the sharing of official information and the sharing of unofficial information. Maximum likelihood estimation was used. The path model was evaluated based on the standard cut-off values for the model–data fit indices [65]. Furthermore, the significance of indirect effects proposed in RQ1 was tested through bootstrapping (*n* = 5000, bias corrected; see [66]). Unstandardized coefficients and their standard errors are reported in the subsequent section.

Chi-square (*df* = 6, *N* = 832) = 31.91, CFI = 0.99, RMSEA = 0.07, 90% CI RMSEA = [0.04, 0.09], SRRM = 0.017. Covariates, including gender, age, education level, and income level, race/ethnicity, issue involvement, social media information seeking were regressed on all exogenous variables.

## 3. Results

### 3.1. Initial Orientations (O_1_)

**Figure 2:**
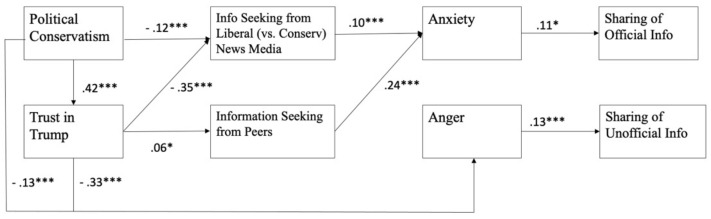
Estimated parameters from the path model. *Note.* *** *p* < 0.001 and * *p* < 0.05. Standardized coefficients are shown in the figure.

The overall model–data fit was good [65], (*df* = 6) = 31.91, SRMR = 0.017, RMSEA = 0.07, 90% CI RMSEA = [0.04, 0.09], and CFI = 0.99 (see Figure 2). The variance explained by the predictors was 0.42 and 0.40 for the sharing of official versus unofficial information, respectively. While H1 assumes that political conservatism relates (a) negatively to seeking information from the liberal versus conservative media and (b) positively to seeking information from peers, our results showed that political conservatism was negatively associated with information seeking from the liberal versus conservative media (*B* = − 0.10, *SE* = 0.02, *p* < 0.001), supporting H1a; however, political conservatism was not associated with peers’ information seeking, disconfirming H1b.

H2 hypothesizes that trust toward Donald Trump relates (a) negatively to seeking information from the liberal versus conservative media and (b) positively to seeking information from peers. There was a positive association between trust toward Donald Trump and peers’ information seeking (*B* = 0.05, *SE* = 0.02, *p* = 0.05) and a negative association between trust toward Donald Trump and seeking information from the liberal versus conservative news media (*B* = −0.21, *SE* = 0.02, *p* < 0.001), supporting both H2a and H2b.

### 3.2. Stimuli (S), Affective Orientations (O_2_), and Response (R)

With respect to whether information seeking through (a) liberal versus conservative media and (b) peers relates positively to anxiety (H3) and whether anxiety relates positively to sharing both (a) official and (b) unofficial information (H4), our results showed support for both H3a and H3b. While information seeking through liberal versus conservative media (*B* = 0.12, *SE* = 0.03, *p* < 0.001) was positively associated with anxiety, information seeking from peers also related positively to anxiety (*B* = 0.21, *SE* = 0.03, *p* < 0.001). Furthermore, anxiety was found to associate with sharing of official information (*B* = 0.12, *SE* = 0.04, *p* < 0.001). Hence, H4a but not H4b was supported.

Subsequently, the relationships between (a) liberal versus conservative media and (b) peers and anger (H5), and the relationships between anger and sharing official and unofficial information (H6) were tested. While information seeking from both liberal versus conservative media and peers did not relate to anger, political conservatism (*B* = −0.14, *SE* = 0.04, *p* < 0.001) and trust in Donald Trump (*B* = −0.26, *SE* = 0.03, *p* < 0.001) were directly associated with anger without going through the two information-seeking variables. Further, our results also showed that anger was associated with sharing unofficial information (*B* = 0.11, *SE* = 0.02, *p* < 0.001), supporting H6b but not H6a.

### 3.3. Mediations

RQ1 asks the extent to which information-seeking behaviors affect sharing of official and unofficial information through various mediating mechanisms. Regarding sharing of unofficial information, our results supported the mediating role of anger in the effects of audiences’ predispositions on the sharing of unofficial information. Both political conservatism and trust toward Donald Trump affected the sharing of unofficial information through anger: indirect effect = −0.02, *SE* = 0.005, *p* < 0.01 for political conservatism and indirect effect = −0.03, *SE* = 0.007, *p* < 0.001 for trust toward Donald Trump.

For the sharing of official information, our results supported the mediating role of anxiety in the effects of information seeking on the sharing of official information. Namely, information seeking from both liberal versus conservative media (indirect effect = 0.015, *SE* = 0.006, *p* < 0.05) and peers (indirect effect = 0.026, *SE* = 0.009, *p* < 0.01) led to anxiety, which in turn affected the sharing of official information.

Last, the full sequence of O-S-O-R was examined by testing the indirect route from political conservatism to liberal (vs. conservative) media information seeking, to anxiety, and ultimately to the sharing of official information. The result was significant (indirect effect = −0.002, *SE* = 0.001, *p* = 0.05). Namely, the more conservative a respondent was, the less information the person sought from liberal versus conservative media, the lower level of anxiety the person experienced, and the less they shared official information.

## 4. Discussion

This study examined the mechanisms driving the sharing of both official and unofficial information in a pandemic. The consideration of both sharing of official and unofficial information in a single model distinguishes this research from other models predicting information sharing. By illuminating the different affective mechanisms as well as identifying the different initial orientations and stimuli that drive the sharing of two types of information via the O-S-O-R model [6], our results contribute to the understanding of how scholars and practitioners can promote official content to enhance the effectiveness of communication in times of a politicized health crisis.

### 4.1. Affective Mechanisms

Recognizing the mediating effects of affective orientations (O_2_) on the consequences of information sharing from both official and unofficial sources, anxiety and anger were found to trigger distinct information-sharing behaviors. This finding supports the extant theorizing on emotions and information behaviors in crises or issues involving risk implications [51]. While both affective orientations are negative in nature, anxiety affected the sharing of official information, while anger toward the U.S. governmental response affected the sharing of unofficial information. These results support the literature regarding how the two distinct emotions can drive different behavioral outcomes [39].

While the literature on the effects of anxiety on information sharing has mixed results [45], our findings add support to the positive relationship between anxiety and official information sharing [46] in a pandemic, in which anxiety does not necessarily mean a full withdrawal of engagement. The lack of full information and personal control over the pandemic might have led people to cope with their stress by relying on professional health authorities. As suggested by Marcus et al. [39], threatening circumstances are likely to drive anxiety. When faced with a threat such as new to humankind such as COVID-19, individuals who experience a lost in personal control [44] share information as a protective behavior [50] when they are motivated to solve the problem [67]. Our findings add support to the positive relationship between anxiety and information sharing [46,48]. More importantly, this positive relationship was found to rely only on official information rather than unofficial information as a strategy. As people cope with anxiety about a new (unfamiliar) pandemic that could be lethal, they tend to communicate with others they know via sharing information from authoritative, official sources even though the pandemic situation is highly politicized. These sources allow the intake of information from the major health authorities (CDC, NIH, and FDA) responsible for containing the pandemic in the United States.

Moreover, when there is a target to blame [49,50], in this case the government, anger can trigger the sharing of unofficial information for two reasons. First, such a sharing behavior can represent a means to express anger [52]. Second, from a functional perspective, such a behavior can supply any information not already in the authorities’ discourse for the angry public to resolve the undesired situation [54]. Behaviors to advocate for information missing from the mainstream discourse make emotion an “approach emotion”, which is associated with mobilizational actions [50]. When one is angry at a crisis manager (i.e., the government), such anger can drive both the acceptance as well as dissemination of unofficial information [53]. Our findings echo this line of health crisis literature [52], such that people who are angry with the government’s response to the pandemic tended to share negative, often unofficial, information to discredit and challenge the government’s action. In short, while anxiety drives the sharing of more credible, official information, encouraging more top-down flow of crisis information, anger drives the sharing of unofficial information, encouraging more information among citizens, or even adding new crisis discourses to the public domain.

### 4.2. Initial Orientations

As anger is likely to be triggered when there is someone or something to blame in a crisis, interventions to reduce the sharing of unofficial information could be difficult. This is particularly true when people are already exposed to information that conflicts with their predispositions, such as political orientation and attitudes toward the target of blame. Overall, audiences’ predispositions play a significant role in people’s information-seeking behaviors and, in turn, their perceptions of the pandemic and information-sharing behaviors related to COVID-19. Given that some conservative politicians downplayed the threat of COVID-19 [26], Americans who trusted Trump might not consider the disease a health priority and thus might turn less to liberal (vs. conversative) information to avoid exposure to information that does not align with their beliefs. These findings not only align with studies which found exposure to traditional news media to be associated with fewer misperceptions regarding COVID-19 [68], but also add support to the selective exposure hypothesis [28].

### 4.3. Stimuli

While most studies have found information seeking to be a key predictor of information sharing [30,69], our findings found a link between the use of liberal relative to conservative news media and official information sharing via anxiety. The consumption of liberal news was likely to create exposure to more official information published by CDC, NIH, and FDA, as well as higher awareness of the potential risks of COVID-19. According to Duffy et al. [4], people who perceive a higher level of risk are expected to share more information as they will likely perceive information, official or not, to be useful and relevant to their friends and family members. As liberal media consumption was found to be associated with higher levels of anxiety [59], the increase in related risk perceptions could trigger anxiety among readers. By sharing information to others, one can protect people in one’s social networks and combat the pandemic by encouraging others to cooperate with the official response measures. It is therefore not surprising to find that liberal (vs. conservative) news media consumption triggered information sharing via anxiety. Our findings contribute to the literature on the O-S-O-R model and health crisis communication.

### 4.4. Implications

In general, the O-S-O-R model suggests that information sharing should be studied without lumping different kinds of information into one sharing variable. By integrating the risk communication and political communication literature, our findings provide two theoretical implications. First, when facing a pandemic that is severe and politicized, people engage in different affective mechanisms to share distinct types of information. Second, given that the literature on online information engagement has so far probed the concept of online information sharing as a unified construct, examining the sharing of official versus unofficial information as distinct constructs is necessary and valuable.

Our study also revealed several practical insights for pandemic communication management. First, to facilitate the positive implications of official information on social media, communication professionals should educate the public about how to form accurate risk perceptions in a complex and saturated information environment. Communication professionals can also provide key opinion leaders (e.g., famous social media influencers, credible political elites, and favorable celebrities) with credible information and encourage them to share such beneficial information with their own networks [70]. Second, an appropriate amount of anxiety may benefit public health by fighting COVID-19 on the societal level due to people’s increased sharing of official health information, even though it is unwise to purposefully elicit anxiety. Communication managers can inform individuals on ongoing pandemic management via accurate and transparent information to maintain a certain level of anxiety, which in turn drives official information sharing. Such an insight could be useful in understanding the seeking and sharing of pandemic information during the pandemic, including the vaccine information that came later in 2021. Finally, crisis specialists and government communication managers should be aware of and act to minimize the anger resulting from poor or indifferent crisis actions and communication to be experienced by audiences in order to discourage unofficial information sharing, especially malicious rumor mongering.

### 4.5. Limitations

Several limitations of this study need to be addressed. First, there might be other channels of information seeking that could be included in the current model, such as health websites. Although information seeking from social media was controlled for and liberal news media and peers did cover the majority of channels for seeking information, future studies should take more information-seeking platforms into account. Second, the data were collected in the United States during the COVID-19 pandemic. The extent to which the findings from this specific context can apply to other scenarios, crises, and countries demands further investigation. Third, while a model was generated with variables placed sequentially in line with extant theorizing, this study utilized a cross-sectional design, and causation cannot be guaranteed. Studies with panel data should be implemented to complement the current findings. Finally, respondents may not have accurately reported their frequency of official and unofficial information sharing. In fact, they might not be willing to report sharing unofficial due to social desirability. Future research should gather actual behavioral data to complement current findings.

## 5. Conclusions

Despite the limitations, this study examined the different mechanisms accounting for the sharing of unofficial and official information during a pandemic. Borrowing from the O-S-O-R model, our findings highlight not only the different roles of anger and anxiety but also the importance of audiences’ predispositions in driving information sharing in cyberspace. In particular, anger toward the U.S. government’s outbreak response was found to associate with unofficial information sharing, while anxiety was associated with official information sharing. Our finding on the relationship between anger and unofficial information sharing potentially contributes to the research on misinformation consumption and sharing by highlighting the roles of particular discrete emotions in driving unofficial information sharing. The findings also reveal the need to investigate different kinds of information sharing given the mix of government–public communication and citizen-to-citizen communication in the current media ecosystem.
